# Editorial: Advancement in kidney care: targeted delivery and precision medicine approaches

**DOI:** 10.3389/fneph.2026.1977844

**Published:** 2026-09-18

**Authors:** Domenico Cerullo, Sonia Fiori, Manuel Alfredo Podestà

**Affiliations:** 1 Department of Molecular Medicine, Istituto di Ricerche Farmacologiche Mario Negri IRCCS, Bergamo, Italy; 2 Unit of Nephrology, Dialysis and Renal Transplantation, Fondazione IRCCS Ca' Granda Ospedale Maggiore Policlinico, Milan, Italy

**Keywords:** biomarkers, chronic kidney disease, immunotherapy, kidney transplantation, precision medicine

Chronic kidney disease (CKD) remains one of the most pressing challenges in global health, affecting more than 10% of the world’s population and contributing significantly to morbidity, mortality and healthcare burden. Despite decades of research and therapeutic advances, CKD continues to progress in many patients, eventually leading to kidney failure and the need for dialysis or transplantation. While diabetes and hypertension account for most cases, a significant proportion of patients suffer from conditions that are less responsive to conventional approaches, including glomerular diseases, immune-mediated nephropathies and rare genetic disorders. These clinical realities highlight the urgent need for innovative strategies capable of slowing, stopping or even reversing disease progression.

This Research Topic was conceived to address these unmet needs by bringing together contributions exploring novel therapeutic targets, emerging diagnostic tools and cutting-edge technologies. The studies included in this collection reflect a rapidly evolving landscape in which advances in immunology, molecular biology, and systems medicine are reshaping traditional paradigms of kidney care.

A key theme emerging from this collection is the growing role of immunomodulation in complex renal conditions. In kidney transplantation, balancing immunosuppression to prevent rejection while preserving host defences remains a major challenge. Gregorini et al., described their successful clinical experience with extracorporeal photopheresis (ECP), in a highly complex kidney transplant recipient with a history of previous graft loss, BK-virus-associated nephropathy, profound alloimmunization and thrombotic complications, which precluded standard therapeutic strategies. ECP provided a personalized immunomodulatory approach capable of rebalancing immune responses without further compromising antiviral immunity. This approach illustrates how immune responses can be recalibrated rather than broadly suppressed, offering a promising option when conventional immunosuppressive adjustments are insufficient or contraindicated. Beyond its therapeutic implications, this case exemplifies how precision medicine in transplantation relies on individualized immunomodulation guided by the patient’s unique immunological profile.

In this regard, increasing attention is being directed toward innovative cellular and immune-engineering approaches. Mesenchymal stromal cell-based therapies have demonstrated promising immunomodulatory and tissue-reparative properties in organ transplantation and may contribute to the induction of graft tolerance while reducing the burden of long-term immunosuppression (Fiori, Remuzzi e Casiraghi, 2020). At the same time, emerging technologies such as bi-specific autoantigen-Tcell engagers seek to selectively eliminate autoreactive B cells by redirecting cytotoxic Tcells toward pathogenic immune populations (Perico et al., 2024). Together, these approaches exemplify the ongoing shift toward precision immunotherapy, in which pathogenic immune responses can be modulated with greater specificity and potentially fewer off-target effects.

Parallel to this, the refinement of pharmacological therapies in immune-mediated kidney diseases continues to advance. The introduction of second-generation calcineurin inhibitors represents a significant step forward in the treatment of lupus nephritis, a condition historically associated with high morbidity and progression to kidney failure. Within this Research Topic, Palmer et al. provide additional insight into the renal safety of second-generation calcineurin inhibition through a *post hoc* analysis of a small subgroup from AURORA 1 trial. Authors demonstrated that voclosporin did not increase biomarkers of kidney injury or fibrosis despite its calcineurin-inhibitory mechanism, also among patients who experienced an early decline in eGFR. These findings are particularly relevant given the longstanding concerns surrounding calcineurin inhibitor-associated nephrotoxicity and further support the favorable renal safety profile of voclosporin.

Improving CKD outcomes depends not only on therapeutic innovation but also on earlier and more accurate detection of kidney dysfunction. In this context, renewed attention to urine biochemistry offers a compelling and pragmatic approach. Maciel, in his perspective article, revisits the diagnostic value of urinary electrolytes, proposing a dynamic interpretation that moves beyond the traditional distinction between “pre-renal” and “intrinsic” acute kidney injury (Maciel, 2025). By focusing on parameters such as urinary sodium concentration and fractional potassium excretion, this approach provides insight into renal microcirculatory stress and early functional impairment. This perspective is particularly relevant given the limitations of serum creatinine, which remains a delayed and often insensitive marker of kidney injury. Changes in urinary electrolyte patterns may precede creatinine elevation by several days, offering a valuable window for early intervention. Reframing urine biochemistry as a real-time indicator of renal physiology supports a more dynamic and physiology-driven model of kidney monitoring, with potential applications extending beyond acute injury to the early detection of subclinical processes that contribute to CKD progression.

Progression to end-stage kidney disease induces profound systemic consequences that become particularly evident in patients requiring maintenance hemodialysis. In this context, beyond the well-described cardiorenal axis, Yu et al. review emerging evidence on the kidney–gut–brain axis, highlighting how gut dysbiosis, uremic toxins, chronic inflammation, and blood–brain barrier dysfunction contribute to cognitive impairment and neuropsychiatric complications. Beyond providing a mechanistic framework, the review highlights the potential of microbiome-derived biomarkers and targeted interventions to support more personalized approaches for patients with advanced kidney disease.

Taken together, the contributions included in this Research Topic highlight a rapidly evolving nephrology landscape in which a deeper understanding of disease mechanisms is increasingly translating into more precise and personalized approaches to diagnosis and treatment. From immune recalibration in transplantation and targeted immunotherapies in immune-mediated kidney diseases to innovative biomarkers and systems-based perspectives, these studies collectively emphasize the need to move beyond traditional disease classifications toward biologically informed strategies tailored to individual patients.

The next phase of kidney medicine will require bridging the gap between scientific discovery and clinical implementation. This will depend on robust validation of emerging therapies and biomarkers, integration of molecular and clinical information into decision-making pathways, and collaborative efforts across disciplines. Ultimately, the goal is to transform CKD management from a predominantly reactive approach focused on slowing progression into a proactive model aimed at early intervention, disease modification, and preservation of kidney function for a broader and more diverse patient population.

